# Giant Extragastrointestinal Mesenteric Stromal Tumor: A Case Report and Literature Review of a Rare Entity With Nonspecific Symptoms

**DOI:** 10.7759/cureus.78135

**Published:** 2025-01-28

**Authors:** Dario E Medina-Muñoz, Roberto Morales-Ramírez, César J Treviño-Arizmendi, Carlos Pacheco-Molina, Francisco Vásquez-Fernádez, Eduardo Navarro-Bahena, Marco A Treviño-Lozano, Gerardo E Muñoz-Maldonado, Rodrigo H Girón-Cuestas, Alvaro Barbosa-Quintana

**Affiliations:** 1 General Surgery, Dr. José Eleuterio González University Hospital at the Autonomous University of Nuevo León, Monterrey, MEX; 2 Pathology and Laboratory Medicine, Dr. José Eleuterio González University Hospital at the Autonomous University of Nuevo León, Monterrey, MEX

**Keywords:** abdominal mass, extragastrointestinal stromal tumor, mesenteric tumor, oncology, surgical resection

## Abstract

Extragastrointestinal stromal tumors (EGISTs) are rare mesenchymal neoplasms with a low frequency compared to gastrointestinal stromal tumors (GISTs). EGISTs share histological and immunohistochemical features with GISTs but occur outside the gastrointestinal tract, commonly in the mesentery or omentum. We report the case of a 52-year-old male presenting with a large asymptomatic abdominal mass. Imaging revealed a semi-solid-cystic tumor measuring 22 x 14 x 21.8 cm. Exploratory laparotomy confirmed an EGIST originating in the mesentery, which was successfully resected without complications. Histopathology showed high mitotic activity, *CK117* positivity, and areas of necrosis. Postoperative recovery was uneventful, and the patient remains under follow-up. To the best of our knowledge, this case represents the first documented EGIST at José Eleuterio González University Hospital and one of the largest reported in Mexico. EGISTs remain poorly understood, and early surgical intervention remains the cornerstone of treatment. Further research is essential to develop tailored guidelines for their management.

## Introduction

Gastrointestinal stromal tumors (GISTs) are rare, comprising less than 1% of all intestinal tumors, with an incidence of approximately 1 per 6.8 million people in the United States. The most common sites of origin include the stomach (60%), small intestine (30%), and colon (10%) [1]. These tumors arise from mesenchymal cells, specifically interstitial cells of Cajal, and are considered to have malignant potential in two-thirds of all cases. The most commonly used criteria to assess prognosis and survival include tumor size, cellularity, nuclear pleomorphism, and mitotic rate [2].

Extragastrointestinal stromal tumors (EGISTs) are exceedingly rare, representing only 5%-10% of all GISTs [3]. They share the same histological and biological immunohistochemical features as GISTs and demonstrate a similar behavior. Immunohistochemical tests consistently show *CD117 *expression, a *CKIT *protein, in cases of EGIST. Imaging protocols for these tumors include computed tomography (CT), magnetic resonance imaging (MRI), and positron emission tomography [4].

Discovered in 2000 by Reith and collaborators, these abdominal neoplasms behave like standard GISTs [5]. Few reported cases suggest a slight predominance in women having tumor sizes of less than 12 cm, and origins in the mesentery or omentum [6]. 

EGISTs are the least commonly reported type of GIST. In Mexico, only three cases originating in the mesentery have been documented [2]. Most EGIST cases are reported as malignant, with limited studies available on their incidence, pathogenesis, prognosis, and prognostic biomarkers [1]. Typically, these tumors are assessed using the same criteria as GISTs, considering factors such as tumor size, mitotic rate, and the presence of tumor necrosis.

## Case presentation

**Figure 1:**
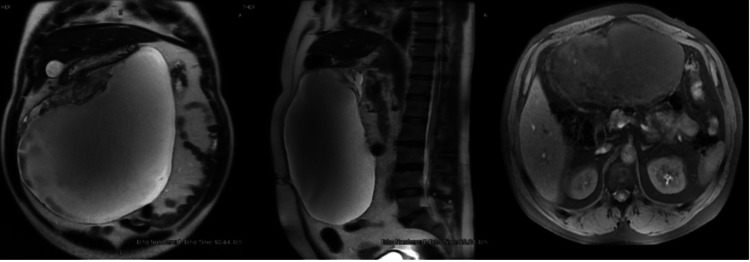
Preoperative magnetic resonance imaging.

We present the case of a 52-year-old male patient with no relevant pathological history who presented with a slowly growing, asymptomatic abdominal mass of six months’ duration. The patient sought medical evaluation after experiencing early satiety, increased abdominal girth, and moderate exertional dyspnea. A contrast-enhanced abdominal CT scan revealed a heterogeneous, semi-solid-cystic tumor with an estimated volume of 3,890 cc and dimensions of 22 x 14 x 21.8 cm. Subsequently, an abdominal MRI study suggested a cystic lesion, raising suspicion for a gastric-origin GIST (Figure 1). 

**Figure 2:**
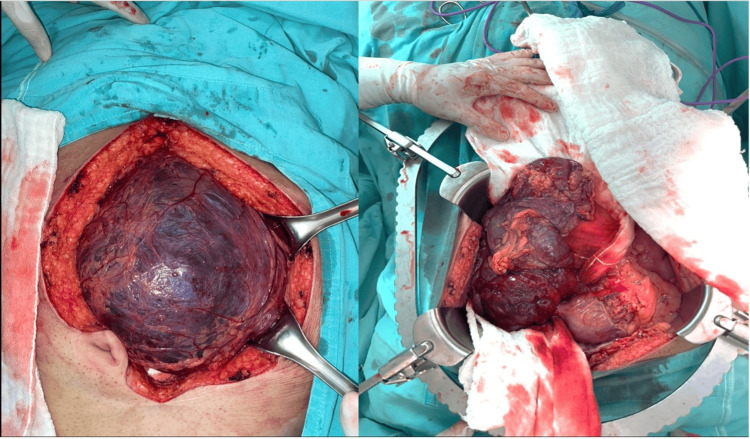
Intraoperative images of a mesenteric-dependent extragastrointestinal stromal tumor cyst.

The patient was evaluated by the general and oncological surgery departments, which decided on an exploratory laparotomy. Intraoperatively, a congestive cystic tumor was identified, which was non-adherent, mobile, and originated from a polyp on the greater curvature of the stomach. However, multiple adhesions to the peritoneal and mesenteric regions were noted (Figure 2).

**Figure 3:**
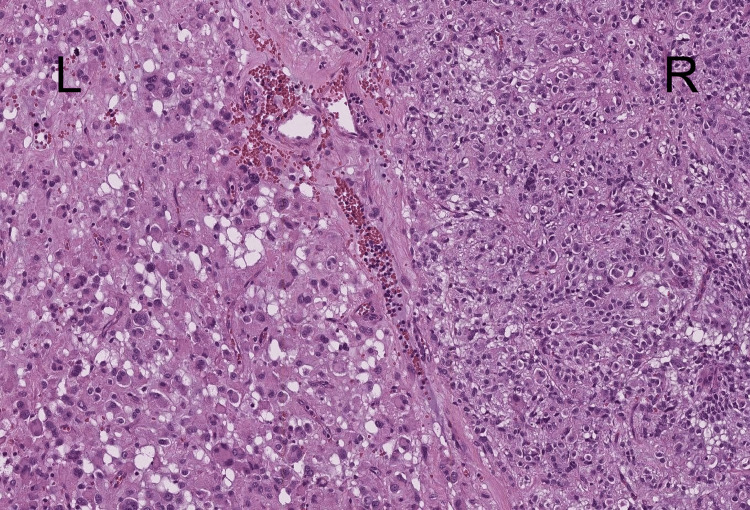
Hematoxylin & Eosin staining. Right, R: Component with epithelioid appearance; Left, L: larger multinucleated cells with hyperchromatic nuclei, marked loss of the nuclear-to-cytoplasmic ratio. Observed at 10x magnification.

Complete resection was achieved. Microscopic pathological examination revealed an abrupt transition from an epithelial neoplasm (on the right side) to a solid pattern of large cells with nuclear-cytoplasmic ratio loss, hyperchromatic features, multinucleation, primitive appearance, and loss of epithelial differentiation (on the left side) (Figure 3). 

**Figure 4:**
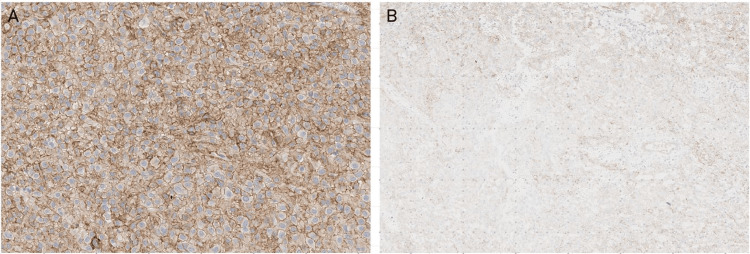
Immunohistochemical markers were performed. A) DOG-1 positive; B) CD117 positive, with lesser expression towards the center of the tumor. Observed at 10x magnification.

Surgical margins were negative. The tumor was identified as a mesenteric-dependent undifferentiated GIST with a high mitotic index, necrosis, and* CK117* positivity, consistent with an EGIST (Figure 4). 

**Figure 5:**
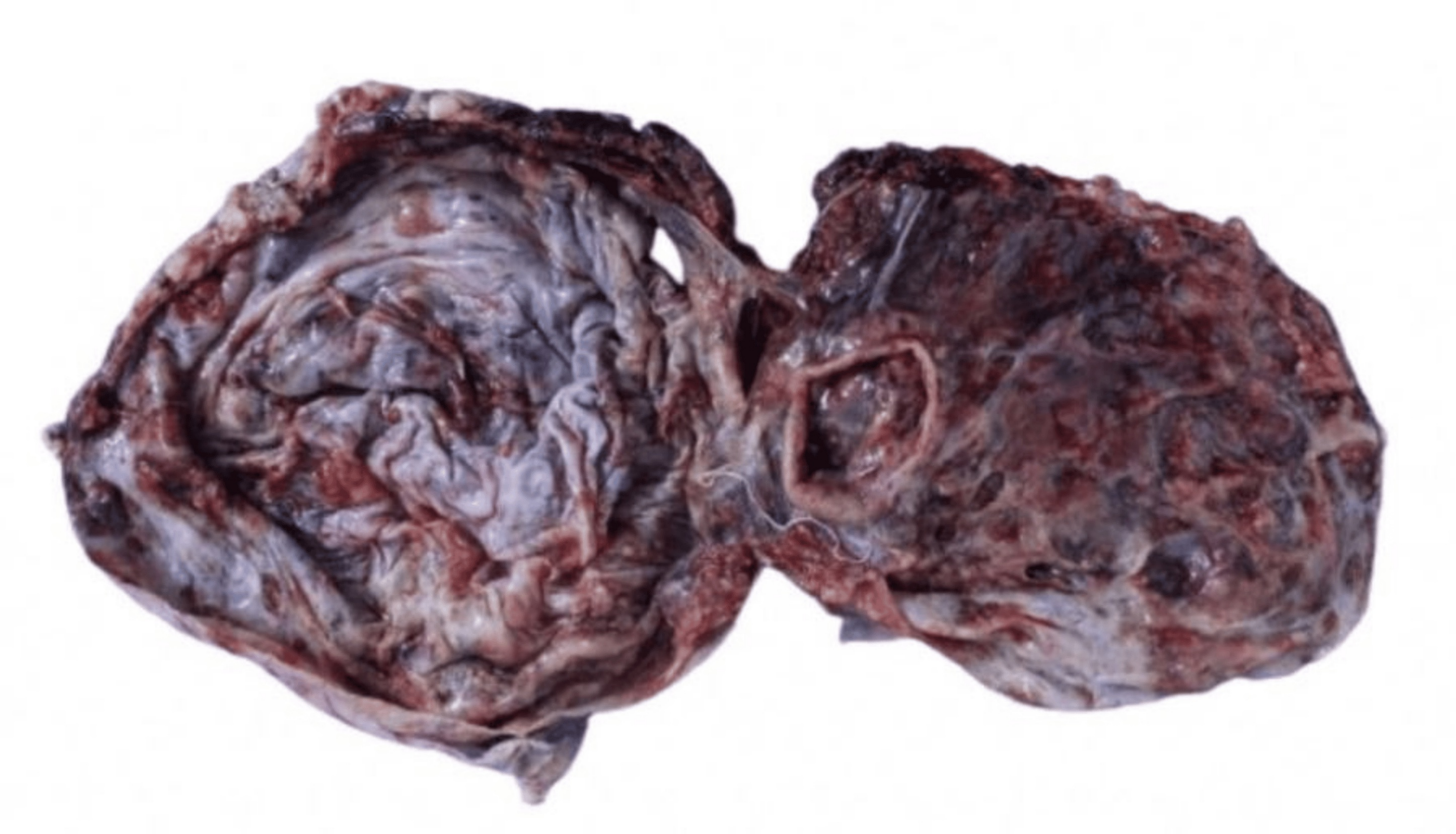
Tumor section. The tumor shows showing a solid-cystic, multiloculated surface with the release of serous fluid Some areas have a solid appearance that alternate between light brown and yellow in color, and these regions have a friable consistency.

Macroscopic analysis indicated a multilocular solid-cystic mass with a serous fluid component and light brown to yellow friable solid areas (Figure 5).

The patient had an uncomplicated postoperative course and was discharged four days after surgery. This represents the first reported case of EGIST at José Eleuterio González University Hospital, Monterrey, Nuevo León. The patient is currently under oncology follow-up and has been informed about the potential for recurrence.

## Discussion

Large mesenteric stromal tumors are considered extremely rare, with the largest case series including 12 to 16 patients over several years, according to retrospective studies. The average age of incidence is 63 years, with tumor size ranging from 0.5 to 44 cm in diameter at the time of diagnosis. Symptoms such as abdominal pain, early satiety, or dyspepsia may arise secondary to compression of adjacent structures. [7]. Other symptoms include hematemesis, melena, or symptomatic anemia [2]. The most commonly observed mutations involve the *KIT tyrosine kinase *or *PDGFRA genes *[6]. Treatment is multidisciplinary, involving complete surgical resection combined with chemotherapy using imatinib [8]. The prognosis of GISTs is determined using the National Institutes of Health classification, with size thresholds of 2 cm for small tumors, 5 cm for medium-sized tumors, and >10 cm for large tumors, with the last associated with a worse prognosis [9].

These tumors are frequently large at the time of diagnosis due to their asymptomatic nature and rapid growth. Giant tumors reaching dimensions of 23x41x38 cm have been reported [6]. Imatinib use in GIST treatment began in 2002 after approval by the FDA, with reported efficacy including partial and complete responses in up to 53.8% of cases. However, 10%-15% of GISTs are considered resistant to imatinib therapy [6]. GISTs are typically exophytic and predominantly solid; some authors propose that tumors with more than 75% cystic components should be classified as cystic GISTs [1]. 

Unfortunately, these tumors are often asymptomatic and are usually diagnosed when they have reached a significant size. Spontaneous hemoperitoneum due to tumor rupture has been reported in some cases [10]. Preoperative Diagnosis is primarily made using imaging studies, including contrast-enhanced CT and MRI. Common differential diagnoses include gastric duplication cysts,mucin-producing tumors, pancreatic pseudocysts, and cystic lymphangiomas [1]. This case serves as an appropriate example of the use of imaging studies, clinical follow-up, and comprehensive pathological analysis in such patients. 

## Conclusions

EGISTs are very rare mesenchymal neoplasms with limited information available on their management. To the best of our knowledge, this is the first documented case of EGIST reported in the literature from José Eleuterio González University Hospital. Additionally, it is among the largest reported cases in Mexico in terms of tumor size, with only a few isolated cases documented in recent years. Currently, there are no specific guidelines to individualize medical or surgical treatment for EGISTs; conventional GIST management guidelines continue to be applied. Retrospective clinical studies are necessary to expand therapeutic options and better address the challenges of treating this rare tumor.
